# Grand Rounds: Nephrotoxicity in a Young Child Exposed to Uranium from Contaminated Well Water

**DOI:** 10.1289/ehp.9707

**Published:** 2007-05-22

**Authors:** H. Sonali Magdo, Joel Forman, Nathan Graber, Brooke Newman, Kathryn Klein, Lisa Satlin, Robert W. Amler, Jonathan A. Winston, Philip J. Landrigan

**Affiliations:** 1 Western University of Health Sciences, College of Osteopathic Medicine of the Pacific, Pomona, California, USA; 2 Department of Pediatrics and; 3 Department of Community and Preventive Medicine, Mount Sinai School of Medicine, New York, New York, USA; 4 School of Public Health, New York Medical College, New York, New York, USA; 5 Department of Medicine, Mount Sinai School of Medicine, New York, New York, USA

**Keywords:** beta-2-microglobulin, drinking water, drinking water standards, groundwater, nephrotoxicity, private wells, uranium

## Abstract

**Context:**

Private wells that tap groundwater are largely exempt from federal drinking-water regulations, and in most states well water is not subject to much of the mandatory testing required of public water systems. Families that rely on private wells are thus at risk of exposure to a variety of unmeasured contaminants.

**Case Presentation:**

A family of seven—two adults and five children—residing in rural northwestern Connecticut discovered elevated concentrations of uranium in their drinking water, with levels measured at 866 and 1,160 μg/L, values well above the U.S. Environmental Protection Agency maximum contaminant level for uranium in public water supplies of 30 μg/L. The uranium was of natural origin, and the source of exposure was found to be a 500-foot well that tapped groundwater from the Brookfield Gneiss, a geologic formation known to contain uranium. Other nearby wells also had elevated uranium, arsenic, and radon levels, though concentrations varied widely. At least one 24-hr urine uranium level was elevated (> 1 μg/24 hr) in six of seven family members (range, 1.1–2.5 μg/24 hr). To assess possible renal injury, we measured urinary beta-2-microglobulin. Levels were elevated (> 120 μg/L) in five of seven family members, but after correction for creatine excretion, the beta-2-microglobulin excretion rate remained elevated (> 40 μg/mmol creatinine) only in the youngest child, a 3-year-old with a corrected level of 90 μg/mmol creatinine. Three months after cessation of well water consumption, this child’s corrected beta-2-microglobulin level had fallen to 52 μg/mmol creatinine.

**Significance:**

This case underscores the hazards of consuming groundwater from private wells. It documents the potential for significant residential exposure to naturally occurring uranium in well water. It highlights the special sensitivity of young children to residential environmental exposures, a reflection of the large amount of time they spend in their homes, the developmental immaturity of their kidneys and other organ systems, and the large volume of water they consume relative to body mass.

Groundwater is the principal source of drinking water for 14–15 million (14%) of the 105.5 million homes in the United States and for approximately 42 million people [Centers for Disease Control and Prevention (CDC) 2003; U.S. Census Bureau 2000; U.S. Environmental Protection Agency (EPA) 2006].

Groundwater is at risk of contamination by a wide variety of industrial pollutants and naturally occurring toxic chemicals. Industrial chemicals that have been identified in ground-water include benzene, methyl *tert*-butyl ether, nickel, perchlorate, perchloroethylene, pesticides, phenol, and trichloroethylene [Agency for Toxic Substances and Disease Registry (ATSDR) 1996, 1997a, 1997b, 1998, 2005b, 2005c; Baker et al. 1978; U.S. EPA 2006]. These contaminants are most commonly found near chemical and pesticide production facilities, hazardous waste sites, roads, and railways. Naturally occurring toxic chemicals that have been documented in groundwater include arsenic, manganese, radon, and uranium (ATSDR 1999, 2005a;U.S. EPA 2006). These materials may be present in especially high concentrations in mining districts, but also occur widely in certain geologic formations, especially in mountainous areas of the United States (U.S. EPA 2006; Walsh 2003).

Private wells that tap groundwater have been associated with episodes of human exposure to toxic chemicals (U.S. EPA 2006). Private wells in the United States are largely exempt from state and federal drinking water regulations, and thus in most states they are not subject to much of the mandatory testing that is required of public water supplies under the provisions of the Safe Drinking Water Act Amendments of 1996 (1996). In most locations, well water is routinely tested only for pH, bacteria, and a small number of chemical contaminants. Uranium is not commonly among the chemicals tested. Because of increasing urban sprawl with continuing movement of populations from urban centers to previously rural areas that lack public water supplies (Frumkin et al. 2004), a growing number of private wells are being drilled in the United States. Unless changes are mandated in current testing requirements, the number of people at risk of exposure to toxic chemicals in groundwater will therefore likely increase.

Exposures to chemical contaminants in groundwater have caused disease and disability in exposed populations. The prevalence and severity of these effects reflect the intensity, the duration, and the developmental timing of exposure. Reported health effects have included diminished intelligence after prenatal exposures to lead and manganese; peripheral vascular disease and skin cancer after childhood exposure to arsenic; and fatal methemoglobinemia after exposure in infancy to nitrates (Ahsan et al. 2006; Campbell 1952; Needleman et al. 1979).

Infants and young children are especially vulnerable to chemical contaminants in drinking water. This heightened vulnerability reflects the disproportionately great water consumption of young children, who drink 7 times as much water per kilogram body weight per day as the average adult (Ershow and Cantor 1989) (Figure 1). It also reflects the inherent biological vulnerability of the young, which is a consequence of their rapid growth and development and their relative inability to detoxify and excrete many exogenous chemicals [National Research Council 1993].

We present a case of a family who was exposed to naturally occurring uranium in groundwater from their private well in Connecticut. Although all family members had evidence of exposure, the only family member with evidence of nephrotoxicity was the youngest child.

## Case Presentation

In September 2000 a family of seven—two adults and five children 3, 5, 7, 9, and 12 years of age—living in a development in rural northwestern Connecticut discovered highly elevated levels of uranium in the drinking water of the home where they had resided for 5 years. The home water was supplied by a private well that tapped groundwater at a depth of approximately 500 feet. The family had used water from this well for cooking, drinking, and bathing from the time that they had moved into the home until discovery of the contamination.

The family first became aware of the possibility of uranium exposure after a neighbor was found to have markedly elevated levels of uranium in her hair. This neighbor had had her hair tested for a range of metals because she was concerned that she had been exposed to mercury from her dental fillings; mercury levels in the neighbor’s hair were not elevated.

After the discovery of elevated levels of uranium in her hair, the neighbor had the water from her well tested for uranium. Her well water was found to contain uranium at a level of 41 pCi/L. Applying the U.S. EPA conversion factor of 0.9 pCi/μg (an estimate based on the ratio of uranium species found typically by the U.S. EPA in well water) this translates to 46 μg/L, a value above the U.S. EPA maximum contaminant level (MCL) for uranium in public water supplies of 30 μg/L (aNational Primary Drinking Water Regulations 2001a). In response to their neighbor’s discovery of uranium in her well water, the index family had their well water tested. Initial testing in a private laboratory (RSA Laboratories, Inc., Hebron, CT) on 28 September 2000 returned a level of 866 μg/L (779 pCi/L), a value also above the U.S. EPA MCL. Repeat measurement by the State of Connecticut Department of Public Health Laboratory (Hartford, CT) on 12 October 2000 returned a level of 1,160 μg/L (1049 pCi/L). Investigation for other contaminants revealed that arsenic was present at a concentration of 0.104 mg/L (above the current U.S. EPA MCL of 0.01 mg/L) (U.S. EPA 2001b) and radium 226 and 228 combined activity was measured at 15.61 pCi/L (above the U.S. EPA MCL for combined activity of 5 pCi/L). The family was advised to immediately stop consuming water from their home well.

### Environmental assessment

Environmental investigation was undertaken by the State of Connecticut Department of Public Health (Hartford, CT). Agency staff collected samples from the index family, as described above, and from other nearby homes in the development where the family resided. The State of Connecticut Department of Public Health Laboratory analyzed the water samples for uranium and radon and air samples for radon.

Environmental assessment revealed that four of 11 homes tested in the development where the index family resided had elevated levels of uranium in their well water. The level of uranium contaminations was quite variable, with very high levels occurring in some homes and almost none in adjoining homes (Table 1).

Geologic assessment by the Connecticut Department of Public Health determined that this housing development had been built in the Appalachian foothills of northern Connecticut and that it sat above the “Brookfield Gneiss,” a metamorphic rock formation common throughout the Appalachian ridges of western New England. Geologic formations similar to the Brookfield Gneiss have been shown to contain concentrated pockets of natural uranium (Robinson and Kapo 2003; Walsh 2003). The Connecticut Department of Public Health considered naturally occurring metals in the Brookfield Gneiss to be the most likely source of the uranium and other minerals detected in the index family’s well water. Review of town and county records and of business directories revealed no evidence of any current or past metal mining or other industrial source of uranium or other toxic metals in the area.

### Clinical assessment

To assess the uranium exposure of family members, a 24-hr measurement of urine uranium was obtained by a pediatric nephrologist in our group (L.S.) from all family members in October 2000, 4 weeks after cessation of well water consumption, an event that had occurred on 28 September 2000 (Table 2). An additional 24-hr collection was obtained from the parents in November 2000, 6 weeks after cessation of well water consumption. At least one urine uranium measurement was found to be elevated in six of the seven family members. Levels ranged from < 1 μg/L to 6.2 μg/L, well above the mean concentration in the U.S. population of 0.009 μg/L (CDC 2005). When adjusted for urinary volume, uranium excretion was 1.1–2.5 μg uranium/24 hr (values above the < 1 μg uranium/24 hr expected in an unexposed population). Although elevated levels of radon, radium, and arsenic were found in the family’s water, biological measures were not pursued because these substances are not known to have chemical nephrotoxic effects. The MCLs for these substances are designed to protect against the elevated risk of cancer that exposure confers.

To assess the possible occurrence of renal tubular injury, measurements were made (by L.S.) in family members of urine beta-2-microglobulin levels (Table 2). Beta-2-microglobulin is a low-molecular-weight (11.8 kD) protein that is freely filtered at the glomerulus and avidly taken up and catabolized by the proximal tubule (Brenner 2004). Elevation of urine beta-2-microglobulin is a nonspecific marker of proximal tubule damage. Elevated beta-2-microglobulin levels (normal reference range in adults is < 120 μg/L) were found in five of the seven family members and ranged from 89 to 530 μg/L, thus suggesting the possible presence of proximal tubular injury. To adjust the beta-2-microglobulin for urine volume and body mass in children, a urinary beta-2-microglobulin excretion rate (micrograms beta-2-microglobulin per gram creatinine) is commonly calculated (Tomlinson 1992). The beta-2-microglobulin excretion rate was normal (< 40 μg/mmol creatinine) in all family members except for the youngest child. In this 3-year-old, who unlike other family members had spent virtually all of her life in the house, the urinary beta-2-microglobulin excretion rate was 90 μg/mmol creatinine, a value more than twice the reported upper limit of normal. Three months after the family had ceased consuming water from the home well (January 2001), this child’s urinary beta-2-microglobulin excretion rate had fallen to 52 μg/mmol creatinine. There was no evidence of other proximal tubule dysfunction, as evidenced (in January 2001) by the absence of glucosuria, phosphate wasting (with normal values for the tubular reabsorption of phosphate), bicarbonate wasting, or metabolic acidosis (Table 3).

Analytic methods for key environmental and biological tests are reported in Table 4.

## Discussion

This case underscores the potential hazards of consuming groundwater from private wells. It emphasizes the need to test drinking water for a wide range of potential contaminants. Specifically, it documents the potential for significant residential exposure to naturally occurring uranium in well water. The case also highlights the special sensitivity of young children to environmental exposures in the home—a reflection of the large amount of time young children spend in their homes, their developmental immaturity, and the large volume of water they consume relative to their body mass.

Uranium is a commonly occurring radioactive mineral. It is found naturally in geologic formations such as the Brookfield Gneiss. In the formation of metamorphic rock, uranium is distributed very unevenly. It typically deposits in areas of low pressure and irregular cracks. Therefore, concentrations can vary significantly within a small area. The level of uranium that appears in drinking water depends on the flow of water through complicated fracture networks within the rock, as well as on the pH, calcium content and other characteristics of groundwater. For these reasons, concentrations of uranium in closely adjoining wells may be quite different, as was seen in this case. This pattern of significant local variability in concentrations of uranium has been observed in various locations across North America (Natural Resources Canada 2005). Given the unpredictability of uranium concentrations in at-risk areas, testing of well water for the presence of uranium at the time of drilling a new well or the sale or transfer of a property with an existing well is a reasonable measure (ATSDR 1999).

Uranium can enter the body via inhalation as well as through consumption of contaminated food or water (Mao et al. 1995). Dermal absorption is seen principally in the instance of military veterans who have been exposed to munitions containing depleted uranium and suffered puncture wounds (Bleise et al. 2003). Ingested uranium is absorbed from the digestive tract and appears initially in the blood, bound to red blood cells. Most is excreted via urine and feces, and experimental studies in humans have shown that about two-thirds of an injected dose of uranium is excreted within the first 24 hr and 75% within 5 days (Taylor and Taylor 1997). Retained uranium accumulates initially in the kidneys and liver and then in the skeleton (Li et al. 2005). Approximately 50–60% of stored uranium in the human body is found in the skeleton (Fisenne and Welford 1986). The biological half-life of uranium in the skeleton is approximately 300 days. The amount of uranium present in skeletal tissue is proportional to cumulative absorption (Hursh and Spoor 1973).

Uranium has the potential to be both chemically and radiologically toxic, but of principal concern in the context of ground-water exposure are the chemical toxic effects of uranium on the kidneys. The most extensive data on the human toxicity of uranium come from studies conducted on workers occupationally exposed in the nuclear industries (Thun et al. 1985); these studies demonstrated increased excretion of beta-2-microglobulin with increasing duration of exposure to uranium. Investigations of Gulf War veterans exposed to depleted uranium did not find clinically significant abnormalities in renal function, but did demonstrate that mean concentrations of microalbumin were significantly elevated in the group exposed to high levels of uranium (Harley et al. 1999; Squibb et al. 2005). There is also evidence that uranium may cause toxic effects in bone (Kurttio et al. 2005).

Within the kidneys, the proximal tubules are the structures principally damaged by uranium (Mao et al. 1995; Zamora et al. 1998). There is no evidence for glomerular injury (Kurttio et al. 2002). Evidence for dose-related proximal tubular injury has been observed after both ingestion and injection of uranium in animal (Diamond et al. 1989; Gilman et al. 1998) as well as in human (Zamora et al. 1998) studies. The histopathologic damage to the proximal tubules is manifest as cytoplasmic vacuolation, interstitial scarring, and destruction of the basal lamina (Gilman et al. 1998).

The pathophysiologic consequences of the proximal tubular injury associated with exposure to uranium include decreased ability to reabsorb water and small molecules, as is evidenced by the presence of elevated levels of the low-molecular-weight protein beta-2-microglobulin in the urine (Kurttio et al. 2002; Mao et al. 1995; Zamora et al. 1998;). Another marker for proximal tubule damage—increased fractional excretion of calcium and phosphate—has been observed to increase in dose-related manner after chronic ingestion of water containing uranium; this change has been observed in the absence of any increase in urinary beta-2-microglobulin to creatinine ratio (Kurttio et al. 2002). There appears to be no clear threshold for these pathophysiologic changes, and they typically become evident before any histopathologic evidence of injury is manifest (Kurttio et al. 2002). The severity of the tubular injury caused by uranium exposure has been shown in rat experiments involving relatively high-dose exposures to range from mild proximal tubular dysfunction to tubular necrosis (Haley 1982).

**Figure 1:**
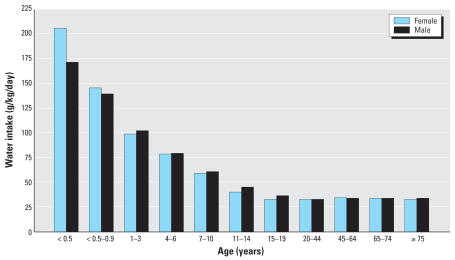
Mean daily intake of total water per unit of body weight by age group and sex. Figure reprinted from Ershow and Cantor (1989), with permission from Life Sciences Research Office.

Although specific studies on the nephrotoxic effects of uranium in children have not been conducted, it is reasonable to assume that children would be at increased risk for adverse effects from exposure compared with adults. Children consume more water and food per kilogram of body weight than do adults (Figure 1) (Ershow and Cantor 1989; National Research Council 1993). Thus children will ingest proportionately greater quantities of any contaminants that are present in the water or food that they consume. For example, the 3-year-old girl in this case series who manifested elevated urinary excretion of beta-2-microglobulin was reported to derive a major portion of her nutritional intake from infant formula that was prepared by mixing powdered formula with contaminated well water.

Terminal differentiation and maturation of the kidneys and other organ systems occur postnatally, and these developing organs are especially vulnerable to the effects of toxic chemical exposures (National Research Council 1993). Recent studies suggest that chronic uranium exposure is associated with increases in blood pressure (Kurttio et al. 2006). The long-term significance of these changes is unclear. However, children’s long future life expectancy further places them at increased risk of delayed adverse health effects that may develop years or decades after exposure in early life to uranium or other chemical contaminants in drinking water.

Because of its radioactivity, concern has arisen about the possible carcinogenicity of uranium. However, the levels of uranium that have been observed to induce nephrotoxicity are much lower than those that increase risk of cancer, and uranium intake from contaminated water has not been associated with increased risk of human cancer (Auvinen et al. 2002, 2005; Boice et al. 2003; Kim et al. 2004; Kurttio et al. 2002). A recent study that examined a cluster of childhood leukemia cases in Fallon, Nevada, found that the town had levels of uranium above or greatly above the maximum contaminant level. However, the children in Fallon with leukemia did not have a higher exposure to uranium than children without leukemia (Seiler 2004).

Although levels of arsenic, radium, and radon were elevated in the index family’s water supply, none of these substances are known to have nephrotoxic effects.

In summary, this case series demonstrates the potential for significant residential exposure to naturally occurring uranium in groundwater. It underscores the hazards of consuming groundwater from untested private wells (U.S. EPA 2006). It confirms previous epidemiologic studies showing that chronic, low-level exposure to uranium in drinking water may result in mild injury to the proximal renal tubule (Kurttio et al. 2002). It highlights the special sensitivity of young children to environmental exposures (National Research Council 1993). Public health organizations should take the unique exposures and the special vulnerability of children into consideration when setting standards for uranium and other chemical contaminants in drinking water.

## Figures and Tables

**Table 1:** Well water uranium in homes, suburban development, northwestern Connecticut, October–November 2000.

Water uranium	Case family (Home 1)	Home 2	Home 3	Home 4	Home 5	Home 6	Home 7	Home 8	Home 9	Home 10	Home 11
pCi/L	1049.00	41.00	0.19	12.50	7.50	12.20	7.50	469.00	14.70	32.00	19.00
μg/L^a^	1165.56	45.56	0.21	13.89	8.33	13.56	8.33	521.11	16.33	35.56	21.11

Testing done by CT DOH Lab.

a Mass calculated from activity measurements using EPA conversion factor of 0.9 pCi/μg.

**Table 2:** Urine measurements of uranium, β2-microglobulin (β2-M), and creatinine.

Subject	Age [years (sex)]	Test date	Uranium in 24 hr (μg/L)	Urine volume in 24 hr (L)	Uranium excretion (μg/24 hr)^a^	Uranium excretion (ng/mmol creatinine)	β2-M (μg/L)^b^	Creatinine (mmol/L)	β2-M excretion rate (μg β2-M/mmol creatinine)^c^
1	3 (F)	Oct 2000 Jan 2001	6.2	0.4	2.5	1,062	532 267	5.84 5.13	90.8 52
2	5 (M)	Oct 2000 Jan 2001	4.9	0.475	2.3	690	100 174	7.1 9.08	14.1 19.2
3	7 (M)	Oct 2000 Jan 2001	< 1	0.7	< 1	NA	140 343	7.4 12.24	18.9 28
4	9 (M)	Oct 2000 Jan 2001	4.1	0.5	2.1	532	90 89	7.7 11.12	11.7 8
5	12 (M)	Oct 2000 Jan 2001	1.2	1.4	1.7	97	280 167	12.4 9.8	22.6 17
6	34 (M)	Oct 2000 Nov 2000	< 1 1.2	0.85 1.1	< 1 1.3	NA	100	18.5	5.4
7	37 (F)	Oct 2000 Nov 2000	1.3 1.1	0.65 1.025	0.8 1.1	92	130	14.1	9.2

Abbreviations: F, female; M, male; NA, cannot be calculated. Oct and Nov 2000 samples are 24-hr collections; Jan 2001 sample is a random sample. Conversion factor: 1,000 ng = 1 μg. β_2_-M excretion rate = urinary β-2-M:creatinine ratio.

a Uranium reference level < 1.0 μg/24 hours unexposed population.

b β_2_-M reference level ≤120 μg/L (adults 21–57 years of age).

c β_2_-M excretion rate reference level in children: < 40 μg β_2_-M/g creatinine.

**Table 3:** Urine analysis and electrolyte measurements of the five children (1–5).

Characteristic	1	2	3	4	5
Age [years (sex)]	3 (F)	5 (M)	7 (M)	9 (M)	12 (M)
Test date	Jan 2001	Jan 2001	Jan 2001	Jan 2001	Jan 2001
Serum [HCO_3_^–^] (mmol)	21.9	23.0	24.4	28.1	27.7
TRP (%)^a^	92	92	95	92	91
PH	6	7	6	7	6.5
Specific gravity	1.015	1.025	1.020	1.025	1.020
Glucose dip	Negative	Negative	Negative	Negative	Negative
Protein dip	Negative	Negative	Negative	Negative	Negative

a TRP (%) = tubular reabsorption of phosphate = [1 – (*U_p_×S_Cr_*)/(*S_p_* *×U_Cr_*)] *×*100, where *U_p_*, *S_Cr_*, *S_p_*, and *U_Cr_* are urine phosphate, serum creatinine, serum phosphate, and urine creatinine concentrations, respectively. Normal values are > 85%.

**Table 4:** Uranium and β2-microglobulin analytic methods.

Analysis	Method
Uranium in water (RSA Laboratories, CT)	Ion exchange separation with alpha-spectrometry detection (APHA 1992)
Uranium and radium in water (Connecticut State Department of Public Health Laboratory)	Gas proportional analysis; EPA Method 908-0 (U.S. EPA 1980)
Uranium in urine (quest diagnostics sent out to Medtox Laboratories, St. Paul, MN)	Inductively coupled plasma/mass spectrometry
β2-microglobulin in urine (quest diagnostics, Medtox Laboratories, St. Paul, MN)	Fixed rate time nephelometry
Creatinine in urine (quest diagnostics, Medtox Laboratories, St. Paul, MN)	Spectrophotometry

## References

[b1-ehp0115-001237] Ahsan H, Chen Y, Parvez F, Argos M, Hussain AI, Momotaj H (2006). Health Effects of Arsenic Longitudinal Study (HEALS): description of a multidisciplinary epidemiologic investigation. J Expo Sci Environ Epidemiol.

[b2-ehp0115-001237] APHA (1992). Method 7500U: Standard Methods for the Examination of Water and Waste Water.

[b3-ehp0115-001237] ATSDR (1996). Toxicological Profile for Methyl *tert*-Butyl Ether (MTBE).

[b4-ehp0115-001237] ATSDR (1997a). Toxicological Profile for Tetrachloroethylene (PERC).

[b5-ehp0115-001237] ATSDR (1997b). Toxicological Profile for Trichloroethylene.

[b6-ehp0115-001237] ATSDR (1998). Toxicological Profile for Phenol.

[b7-ehp0115-001237] ATSDR (1999). Toxicological Profile for Uranium.

[b8-ehp0115-001237] ATSDR (2005a). Toxicological Profile for Arsenic (*Draft for Public Comment*).

[b9-ehp0115-001237] ATSDR (2005b). Toxicological Profile for Benzene (*Draft for Public Comment*).

[b10-ehp0115-001237] ATSDR (2005c). Toxicological Profile for Nickel. Atlanta, GA:Agency for Toxic Substances and Disease Registry.

[b11-ehp0115-001237] Auvinen A, Kurttio P, Pekkamen J, Pukkala E, Ilus T, Salonen L (2002). Uranium and other natural radionuclides in drinking water and risk of leukemia: a case-cohort study in Finland. Cancer Causes Control.

[b12-ehp0115-001237] Auvinen A, Salonen L, Pekkanen J, Pukkala E, Ilus T, Kurttio P (2005). Radon and other natural radionuclides in drinking water and risk of stomach cancer: a case-cohort study in Finland. Int J Cancer.

[b13-ehp0115-001237] Baker EL, Field PH, Basteyns BJ, Skinner GH, Bertozzi PE, Landrigan PJ (1978). Phenol poisoning due to contaminated drinking water. Arch Environ Health.

[b14-ehp0115-001237] Bleise A, Danesi PR, Burkart W (2003). Properties, use and health effects of depleted uranium (DU): a general overview. J Environ Radioact.

[b15-ehp0115-001237] Boice JD, Mumma M, Schweitzer S, Blot WJ (2003). Cancer mortality in a Texas county with prior uranium mining and milling activities**, 1950–2001. J Radiol Prot.

[b16-ehp0115-001237] Brenner B (2004). Brenner and Rector’s The Kidney.

[b17-ehp0115-001237] Campbell WA (1952). Methaemoglobinaemia due to nitrates in well-water. BMJ.

[b18-ehp0115-001237] CDC (2003). Drinking Water: Private Well Resources. Atlanta, GA:Centers for Disease Control and Prevention.

[b19-ehp0115-001237] CDC (2005). National Report on Human Exposure to Environmental Chemicals. Third Report.

[b20-ehp0115-001237] Diamond GL, Morrow PE, Panner BG, Gelein RM, Baggs RB (1989). Reversible uranyl fluoride nephrotoxicity in the Long Evans rat. Fundam Appl Toxicol.

[b21-ehp0115-001237] Ershow AB, Cantor KP (1989). Total Water and Tapwater Intake in the United States: Population-based Estimates of Quantities and Sources.

[b22-ehp0115-001237] Fisenne IM, Welford GA (1986). Natural U concentrations in soft tissues and bone of New York City residents. Health Phys.

[b23-ehp0115-001237] Frumkin H, Frank L, Jackson R (2004). Urban Sprawl and Public Health: Designing, Planning, and Building for Healthy Communities.

[b24-ehp0115-001237] Gilman AP, Villeneuve DC, Secours VE, Yagminas AP, Tracy BL, Quinn JM (1998). Uranyl nitrate: 91-day toxicity studies in the New Zealand white rabbit. Toxicol Sci.

[b25-ehp0115-001237] Haley DP (1982). Morphologic changes in uranyl nitrate-induced acute renal failure in saline- and water-drinking rats. Lab Investig.

[b26-ehp0115-001237] Harley H, Foulkes EC, Hilborne LH, Hudson A, Anthony CR (1999). A Review of the Scientific Literature As It Pertains to Gulf War Illnesses: Vol. 7, Depleted Uranium, MR-1018/7-OSD; RAND.

[b27-ehp0115-001237] Hursh JB, Spoor NL, Hodge HC, Stannard JN, Hursh JB (1973). Data on man. Handbook of Experimental Pharmacology.

[b28-ehp0115-001237] Kim YS, Park HS, Kim JY, Park SK, Cho BW, Sung IH (2004). Health risk assessment for uranium in Korean ground-water. J Environ Radioact.

[b29-ehp0115-001237] Kurttio P, Auvinen A, Salonen L, Saha H, Pekkanen J, Makelainen I (2002). Renal effects of uranium in drinking water. Environ Health Perspect.

[b30-ehp0115-001237] Kurttio P, Harmoinen A, Saha H, Salonen L, Karpas Z, Komulainen H (2006). Kidney toxicity of ingested uranium from drinking water. Am J Kidney Dis.

[b31-ehp0115-001237] Kurttio P, Komulainen H, Leino A, Salonen L, Auvinen A, Saha H (2005). Bone as a possible target of chemical toxicity of natural uranium in drinking water. Environ Health Perspect.

[b32-ehp0115-001237] Li WB, Roth P, Wahl W, Oeh U, Höllriegl V, Paretzke HG (2005). Biokinetic modeling of uranium in man after injection and ingestion. Radiat Environ Biophys.

[b33-ehp0115-001237] Mao Y, Desmeules M, Schaubel D, Berube D, Dyck R, Brule D (1995). Inorganic components of drinking water and microalbuminuria. Environ Res.

[b34-ehp0115-001237] National Research Council (1993). Pesticides in the Diets of Infants and Children.

[b35-ehp0115-001237] Natural Resources Canada (2005). Environmental Geochemistry and Geochemical Hazards.

[b36-ehp0115-001237] Needleman HL, Gunnoe C, Leviton A, Reed R, Peresie H, Maher C (1979). Deficits in psychologic and classroom performance of children with elevated dentine lead levels. N Engl J Med.

[b37-ehp0115-001237] Orloff KG, Mistry K, Charp P, Metcalf S, Marino R, Shelly T (2004). Human exposure to uranium in groundwater. Environ Res.

[b38-ehp0115-001237] Robinson GR, Kapo KE (2003). Generalized Lithology and Lithogeochemical Character of Near-Surface Bedrock in the New England Region. U.S. Geological Survey Open-File Report.

[b39-ehp0115-001237] Safe Drinking Water Act Amendments of 1996 (1996). Public Law 104–182.

[b40-ehp0115-001237] Seiler RL (2004). Temporal changes in water quality at a childhood leukemia cluster. Ground Water.

[b41-ehp0115-001237] Squibb KS, Leggett RW, McDiarmid MA (2005). Prediction of renal concentrations of depleted uranium and radiation dose in Gulf War veterans with embedded shrapnel. Health Phys.

[b42-ehp0115-001237] Taylor DM, Taylor SK (1997). Environmental uranium and human health. Rev Environ Health.

[b43-ehp0115-001237] Thun MJ, Baker DB, Steenland K, Smith AB, Halperin W, Berl T (1985). Renal toxicity in uranium mill workers. Scand J Work Environ Health.

[b44-ehp0115-001237] Tomlinson PA (1992). Low molecular weight proteins in children with renal disease. Pediatri Nephrol.

[b45-ehp0115-001237] U.S. Census Bureau Profile of Selected Housing Characteristics—2000.

[b46-ehp0115-001237] U.S. EPA (1980). Gas Proportional Analysis. EPA Method 908-0. EPA-600/4-80-032.

[b47-ehp0115-001237] U.S. EPA (U.S. Environmental Protection Agency) (2001a). National Primary Drinking Water Regulations. 40CFR141.1.

[b48-ehp0115-001237] U.S. EPA (U.S. Environmental Protection Agency) (2001b). National Primary Drinking Water Regulations; Arsenic and Clarifications to Compliance and New Source Contaminants Monitoring.

[b49-ehp0115-001237] U.S. EPA (U.S. Environmental Protection Agency) (2006). Private Drinking Water Wells.

[b50-ehp0115-001237] Walsh GJ (2003). Open-File Report 09-487. Bedrock Geological Map of the New Milford Quadrangle, Litchfield and Fairfield Counties, Connecticut.

[b51-ehp0115-001237] Zamora ML, Tracy BL, Zielinski JM, Meyerhof DP, Moss MA (1998). Chronic ingestion of uranium in drinking water: a study of kidney bioeffects in humans. Toxicol Sci.

